# Outcomes after Frey’s procedure for chronic pancreatitis: a 8-year single-center experience in Colombia

**DOI:** 10.1186/s12893-022-01839-x

**Published:** 2022-12-12

**Authors:** Juliana González, Daniela Ayala, Nicolás Caballero, Carlos Eduardo Rey Chaves, Danny Conde, Juan Carlos Sabogal Olarte

**Affiliations:** 1 School of Medicine, Universidad del Rosario, Bogotá, Colombia; 2 Hospital Universitario Mayor Méderi, Bogotá, Colombia; 3 School of Medicine, Pontificia Universidad Javeriana, Bogotá, Colombia; 4 111011 Bogotá D.C, Colombia

**Keywords:** Chronic pancreatitis, Frey procedure, Pancreatojejunostomy, Pancreatic surgery, Pancreatic cancer, Abdominal pain, Follow-up

## Abstract

**Background:**

Chronic pancreatitis is an inflammatory disease characterized by irreversible morphological changes due to chronic pancreatic fibrosis. The treatment goals are to relieve pain, preserve function, and prevent further pathological consequences. Endoscopic treatment, surgery, or both are options for untreatable pain or suspected malignancy. Frey procedure is a reasonable surgical intervention because of its hybrid character, combining resection and drainage. Unfortunately, there is limited information about the outcomes of this procedure in Latin America, and few cases described in Colombia. This study aims to describe the experience of a pancreatic surgery reference center in the management of patients undergoing Frey’s surgery for chronic pancreatitis.

**Methods:**

A retrospective review of a prospectively collected database of patients who underwent a Frey procedure due to chronic pancreatitis between January 2014 to February 2022 in a hospital in Bogotá, Colombia, was made. A demographic, clinical, and postoperative outcome description was performed. Mann–Whitney Willcoxon test was performed between operative variables and long-term outcomes.

**Results:**

Eighteen patients met the inclusion criteria. 55.5% of patients were male. Chronic pancreatitis etiology in most cases (83.3% n = 15) was idiopathic. The median duration of symptoms and chronic pancreatitis diagnosis before surgery was 6.15 months (IQR 5;97). Overall morbidity was 38.88%. One patient died at 30 days of follow-up. The median follow-up time was 42.5 (IQR 19;65 months). The median pain reduction was 3 points according to the visual analog score. Six patients were diagnosed with malignant conditions after surgery (mean 27.8 ± 7.5 months). Wirsung’s duct size was statistically related with malignancy presentation after Frey’s procedure (Z = 2.54; P = 0.01).

**Conclusion:**

According to our data, Frey’s procedure remains safe and feasible, with acceptable outcomes in terms of pain relief and pancreatic function. The study confirms the importance of a longstanding follow-up due to an inherent risk of pancreatic malignancy. Our data suggest that pancreatic duct size could be related with the malignancy diagnosis after Frey’s procedure; however, further prospective studies with a larger sample size would be helpful to confirm these results.

## Background

Chronic pancreatitis (CP) is a progressive inflammatory disease characterized by complex irreversible morphologic changes in the pancreatic parenchyma (ex. edema, inflammation, necrosis, and fibrosis) that lead to a negative impact on the quality of life [1, 2]. It is an uncommon disease with a reported annual incidence of 4–14 cases per 100,000 individuals worldwide [3]. Epidemiological knowledge of CP in Latin America and particularly in Colombia remains scarce [4].

CP’s most common clinical feature is epigastric abdominal pain [5], which radiates to the back, relieves when the patient bends forward, and exacerbates after eating [5–7]. Symptoms of pancreatic insufficiency like steatorrhea, malabsorption, weight loss, and diabetes manifest when approximately 90% of the pancreatic parenchyma is destroyed [8, 9]. Nevertheless, a definitive diagnosis remains challenging and is established based on the patient’s history, clinical presentation, laboratory findings, and imaging [9]. Current evidence-based guidelines recommend computer tomography or magnetic resonance for the first line diagnosis of CP, other options like endoscopic sonography, biopsy or function tests are second line or complementary (strong recommendation, low quality evidence) [10].

The best therapy for CP is still a matter of debate. Current literature suggests initial treatment with conservative measures, such as analgesia, diet changes, pancreatic supplementation, and cessation of alcohol and tobacco [11, 12]; this approach is based mainly on the paradigm that CP is a self-limiting disease [8]. In patients with medical management failure who require therapeutic intervention, the endoscopic approach is the first option before surgery due to its less-invasive nature [13]. Several endoscopic procedures have been described, such as celiac ganglion block, which provides 3–6 months of pain relief, or endoscopic papillotomy with ductal dilation, stent placement, and removal of stones if present, relieving ductal hypertension, that could be the cause of pain [9, 14]. However, approximately 40% to 75% will ultimately require surgery during their disease [8]. Numerous studies have pointed out that early surgery (preferably within 3 years of symptoms beginning) is superior in providing pain relief, reduced risk of pancreatic insufficiency, improvement of quality of life, and the need for further intervention, compared to a step-up approach in which medical therapy is done before endoscopy and, finally, surgery [7, 8].

The most common indications for surgical intervention are debilitating abdominal pain, suspected malignancy, symptoms of pancreatic insufficiency, and local complications such as biliary and duodenal strictures, complex pseudocyst, or failed medical management [9]. The main goals for surgical intervention are to treat effectively, relieve long-lasting pain, reduce morbidity, preserve pancreatic parenchyma, and, therefore, preserve long-term pancreatic function [12] while recognizing that the head of the pancreas constitutes the pacemaker of chronic pancreatitis [8, 12, 15]. Several techniques to treat CP include decompression, surgical resection of the pancreas, or both [12]. The leading operation that decompresses the pancreatic conduct is the lateral pancreaticojejunostomy, which leads to pain relief in almost all short-term patients [11, 12]; however, results have shown recurrence. Furthermore, distal pancreatectomy does not resolve the compressive disease problem in the head of the pancreas, which can deteriorate over time [11, 13]. Pancreatoduodenectomy (PD) has demonstrated reasonable pain control; the indication of PD is a duodenal obstruction or suspected neoplasia [11, 14]; however, it is an effective procedure with a high risk of complications and a high morbimortality [13, 15].

Frey’s procedure is a duodenum preserving head resection combined with lateral pancreaticojejunostomy. This technique has reasonable pain control at long-term follow-up and is associated with a better quality of life, shorter operation time, and lower rate of postoperative complications than other surgical procedures in CP [1, 3, 16]. However, studies in Latin America and Colombia are scarce and fail to characterize its effectiveness and short/long-term post-operative outcomes. Therefore, we aim to describe the experience and outcomes of a pancreatic surgery reference center in managing CP in the population undergoing Frey’s surgery for chronic pancreatitis.

## Methods

### Study population

Through the Institution’s Review Board’s approval and following Health Insurance Portability and Accountability Act (HIPAA) guidelines, the team retrospectively reviewed a prospectively collected database, including all patients over 18 years old who underwent Frey’s procedure between January 2014 and February 2022. Patients with no surgical description, missing data, and follow-up < 1 month were excluded. In addition, ethical compliance with the Helsinki Declaration, current legislation on research Res. 008430-1993 and Res. 2378-2008 (Colombia), and the International Committee of Medical Journal Editors (ICMJE) were guaranteed under our Ethics and Research Institutional Committee (IRB) approval.

### Chronic pancreatitis diagnosis

The hepatobiliary and pancreatic surgery (HPB) assessment group evaluated all patients in external consultation and made the diagnosis according to clinical symptoms such as abdominal pain, pancreatic insufficiency defined as the presence of steatorrhea with fecal analysis of fat absorption, diagnostic of diabetes mellitus, imagenological findings (pancreatic atrophy, pancreatic duct dilatation, and calcifications), or endoscopic assessment according to endoscopic ultrasound criteria (Rosemont criteria).

#### Indications for surgery

The protocol was approved for institutional research and the Ethical committee. Surgery was indicated based on clinical, radiologic, and endoscopy criteria. Surgery was performed in patients in which conservative management failed for pain management and in which endoscopic therapy failed to resolve pancreatolithiasis. Unfortunately, due to economic and health system issues, interventional endoscopy is a limited resource.

### Surgical technique

Under general anesthesia, a midline laparotomy was performed in all cases and conducted a Kocher maneuver to vascular and pancreatic head control. The trans cavity of the omentum is reached by a gastrocolic ligament opening. We dissected the pancreatic head, neck, body, and tail to expose the pancreas fully. Intraoperative ultrasonography was performed to identify the pancreatic duct and begin the pancreatic section until the duct opens. A longitudinal opening of the pancreatic duct in the neck, body, and tail is completed. All pancreatic stones are removed and confirmed by an ultrasound. We finished the resection of the head of the pancreas and sonography vigilance of the bile duct to prevent its injury.

**Fig. 1:**
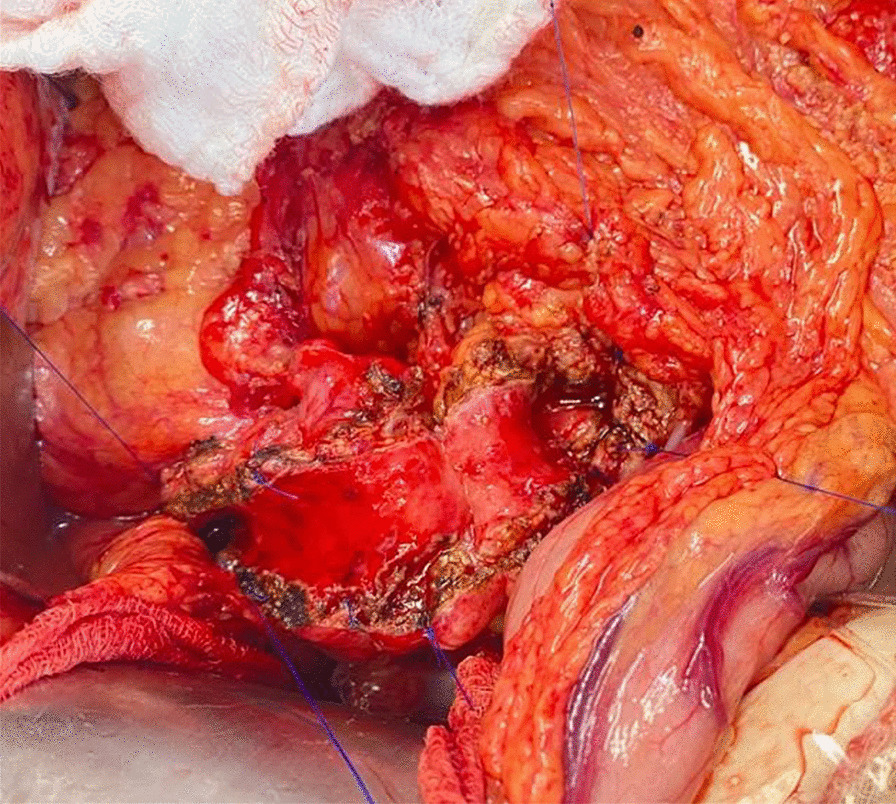
Pancreatic duct at head—body longitudinal opening

**Fig. 2:**
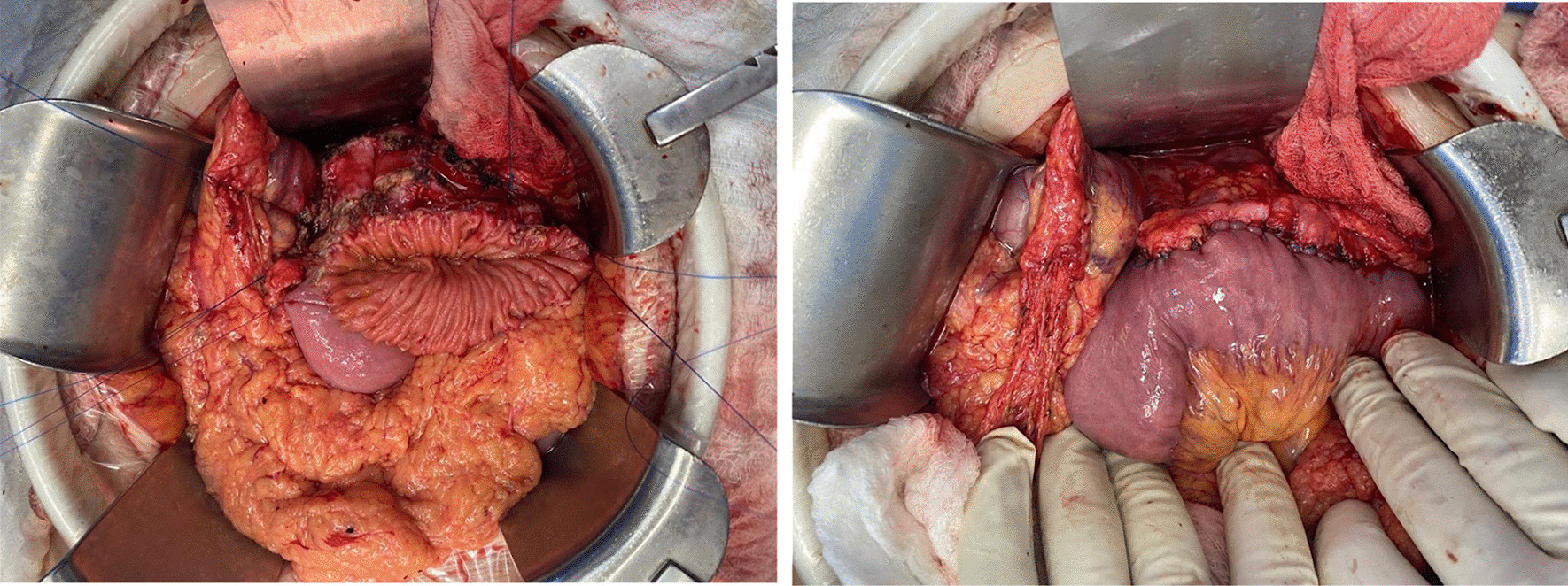
Trans-mesocolic pancreaticojejunostomy

Jejunum is ascended by transmesocolic or antecolic way, jejunum–jejunum anastomosis is performed at 60 cm. a longitudinal opening of the jejunum is made to perform laterolateral pancreatic-jejunum anastomosis. An active drain (Blake or Jackson-Pratt) was left in all cases (Figs. 1, 2).

### Data analysis

Pre-operative data included patient demographics, comorbidities, and surgical and previous endoscopic procedures. Intraoperative and postoperative data included surgical findings, 30 and 90 days morbidity, and mortality. The postoperative pancreatic fistula was defined according to international classification [17] based on drainage amylase analysis at 3 and 5 postoperative days. Overall morbidity was classified according to the Clavien-Dindo classification. Pain assessment was performed using the visual analog scale for pain in the preoperative and postoperative periods. Diabetes mellitus diagnosis was evaluated in both periods as well. Follow-up data was acquired at 8 days, 1, 12, 30, and > 60 months when possible. Pancreatic malignancy related to chronic pancreatitis in the Colombian population was reported according to the National database and pathology reports of our center tracing.

### Statistical analysis

Descriptive statistics were reported in terms of variable nature. Qualitative analysis was performed in terms of frequencies and percentages, while quantitative analysis was done in terms of mean and standard deviations of normally distributed data and medians and interquartile ranges (IQRs) for non-normally distributed data. In addition, a Mann–Whitney Willcoxon test was performed to evaluate the relationship between the pancreatic head and Wirsung’s size with malignancy.

## Results

### Preoperative characteristics

A total of 18 patients underwent Frey’s procedure from January 2014 to 2022. 55.5% of patients were male. The median age was 52.2 (IQR 41;65). The median body mass index (BMI) was 23.1 kg/m^2^ (IQR 18.2;24.92). History of hypertension and type 2 diabetes mellitus (T2DM) was presented in 16.6% (n = 3), a history of smoking in 11.1% (n = 2), and 16.6% of the patients had a history of chronic alcohol consumption. Chronic pancreatitis etiology in most cases (83.3% n = 15) was idiopathic. All patients presented with abdominal pain before surgical intervention, and 83.3% (n = 15) of the population presented with steatorrhea (see Table 1). Previous episodes of acute pancreatitis (AP) were evaluated as well. The mean number of admissions due to AP was 1.16 ± 1.24. The median duration of symptoms and chronic pancreatitis diagnosis before surgery was 6.15 months (IQR 5;97), and in the majority of cases (55.5% n = 10), pancreatolithiasis was observed in magnetic resonance or endoscopic ultrasound. The visual analog scale value median for preoperative pain was 6 points (IQR 4;9). In most of the cases, multimodal pain management was preferred (44.4% n = 8), followed by opioid therapy (11.11% n = 2) and neuromodulators 22.22% n = 4); 50% of the patients required previous endoscopic interventions with Wirsung’s stenting. Preoperative serum markers were evaluated as well. Median white blood cell count was 7.250 Gb/mL (IQR 5.600–12.480). In terms of hemoglobin levels, the median value was 14.25 g/L (IQR 13–15.9). Preoperative albumin median was 3.5 g/dL (IQR 3;4); summarized data is displayed in Table 1.

**Table 1:** Demographic and pre-operative characteristics

Variable	Value
Gender % (n)	
Male	55.56 (10)
Diabetes Mellitus type 2	16.67 (3)
Arterial hypertension	16.67 (3)
Alcoholism	16.67 (3)
Tobacco habit	11.11 (2)
Age median (IQR)	52.5 (41;65)
Body Mass Index median (IQR)	23.1 (18.2;24.9)
Diagnosis time median (IQR)	6.15 (5;1)
Etiology % (n)	
Igg4 related	5.56 (1)
Idiopathic	83.33 (15)
Alcoholic	11.11 (2)
Pancreatolithiasis % (n)	55.56 (10)
Abdominal pain % (n)	100 (18)
Steatorrhea % (n)	85.33 (15)
Previous episodes of acute pancreatitis mean (SD)	1.16 (1.24)
Pre-operative pain score median (IQR)	6 (4;9)
Pre-operative serum analysis	
White blood cell count—Median (IQR)	7.250 (5.600;12.480)
Hemoglobin	14.25 (13;15.9)
Albumin	3.5 (3;4)
Creatinin	0.8 (0.62;1.02)
C-Reactive protein	4.3 (1.5;10)
Previous interventions % (n)	
Endoscopic retrograde colangiopancreatography	22.22% (4)
Pancreatic stenting	27.78 (5)
Pain management % (n)	
Multimodal	44.44 (8)
Opioids	11.11 (2)
Non-steroid anti inflammatory	22.22 (4)
Neuromodulators	22.22 (4)

### Surgical outcomes

The pancreatic head’s median size was 28 mm (IQR 22;65), and the median pancreatic duct diameter was 10 mm (IQR 8;20). Surgical time, including anesthesia induction, was evaluated with a median of 213.5 min (IQR 165;250). The intraoperative bleeding median was 100 cc (IQR 50;300). No patient required intensive care unit admission. The median hospital length stay was 12.6 days (IQR 7;20). After 90 days overall morbidity was 38.88%; the most frequent complication, according to the Clavien-Dindo classification, was type 1 (85.71%). Upper gastrointestinal bleeding was observed in 16.6% (n = 3) of the patients, the postoperative pancreatic fistula was presented in 22.22% (n = 4) of the cases, and all cases were classified as a biochemical leak. Mortality, re-intervention, and readmission rate were 5.5% each (n = 1). Summarized data is displayed in Table 2.

**Table 2:** Operative characteristics and early outcomes

Variable	Value
Pancreatic head size median (IQR)	28 mm (22;65)
Pancreatic duct diameter median (IQR)	10 mm (8;20)
Operative time median (IQR)	213.5 (165;250)
intraoperative bleeding median (IQR)	100 (50;300)
In-hospital stay median (IQR)	12.6 (7;20)
Complication rate % (n)	38.88 (7)
Pulmonary embolism	0 (0)
Pseudoaneurysm	0 (0)
Portal thrombosis	0 (0)
Upper gastrointestinal bleeding	16.67 (3)
Postoperative pancreatic fistula	22.22 (4)
Surgical Site infection	0 (0)
Clavien Dindo	
Type 1	85.71 (6)
Type 5	14.29 (1)
Reintervention rate	5.55 (1)
Mortality rate % (n)	5.55 (1)

### Follow-up

The median follow-up time was 42.5 (IQR 19;65 months). Post-operative diagnosis of T2DM was observed in 22.22% (n = 4) of the cases, but no patient required insulin replacement. 33.3% (n = 6) of patients presented exocrine dysfunction and needed pancreatic enzyme replacement. In the postoperative period, no patient had pancreatolithiasis relapse. The pain was evaluated after 30 days of surgery, with a median 3 points (IQR 1;4) in the visual analog score. A reduction of 50% in pain was observed in the population after surgery (see Table 3).

**Table 3:** Follow-up outcomes

Variable	Value
Follow up median (IQR)	42.5 (19;65)
Postoperative diabetes mellitus diagnosis % (n)	22.22 (4)
Insulin replacement requirement	0 (0)
Exocrine pancreatic dysfunction	33.33(6)
Pancreatolithiasis relapse	0 (0)
Postoperative pain score median (IQR)	3 (1;4)
Postoperative pancreatic malignancy	33.33 (6)
Diagnosis time for malignancy median (SD)	38 (32;75)

### Malignancy follow up

Malignancy was also evaluated according to the national database and institutional tracing; 33.33% (n = 6) of the patients presented pancreatic oncologic conditions related to chronic pancreatitis, and previous histology were revised as well, however, in none of the cases previous data of dysplasia were evidenced. Five of these patients were diagnosed with pancreatic ductal adenocarcinoma and one with neuroendocrine tumor; all patients underwent Whipple’s procedure. The median time between Frey’s procedure and the malignant diagnosis was 38 months (IQR 32;75).

### Statistical analysis

The relationship between pancreatic head and duct size with malignancy was evaluated. A Mann–Whitney Willcoxon test was performed. The test revealed that there was a statistically significant difference in pancreatic duct for patients with malignancy after Frey’s procedure (Z = 2.45 P = 0.01, Exact probability 0.00). Pancreatic head size had no statistical relationship with future malignancy diagnosis (Z − 0.3, P = 0.74, Exact probability 0.76).

## Discussion

Current evidence suggests that surgery is superior to endoscopy in achieving lasting pain relief and that early surgical intervention may help preserve pancreatic function for patients who require therapeutic intervention [18]. Through the evolution of the surgical technique for chronic pancreatitis, Frey’s procedure, due to its hybrid nature, has become a popular surgical intervention with sound pain reduction and low perioperative complications [1, 2].

Overall, we found Frey’s surgery as a safe procedure with a mortality of 5.5% in the 30 days of follow-up; the pain control was reached in 85.3% of the cases, with at least 50% improvement in visual analog score.

Current knowledge about CP’s epidemiology and local clinical features is poorly described in the Latin-American population [18]. Studies in Latin America have reported 1.6 and 3 cases per 100,000 inhabitants per year [19, 20]. The prolonged course of the disease and the complex follow-up of patients lead to late diagnosis [19, 21].

The etiology of CP is still a matter of debate [20, 21]; current guidelines recommend a comprehensive medical history, laboratory evaluation and imaging studies in all patients with suspected CP (Grade 2C, Strong agreement) [22]. Almost 50% of the patients with a confirmed diagnosis of CP had precedents of acute pancreatitis episodes, and in our population has a mean of 1.16 episodes prior to chronic pancreatitis diagnosis; however, there is still no clear association [18, 23]. Alcoholic etiology is recognized as the leading cause of chronic pancreatitis (66%) [23], followed by idiopathic, which reaches up to 29% [23, 24]. In the last decade, the incidence of idiopathic pancreatitis has increased concerning genetic etiology [15, 23, 25]. In our study, 83.3% of the cases (n = 15) were idiopathic, alcoholic etiology was present in 11.11% of the cases (n = 2), and in one case, pathology reported IgG4-related chronic pancreatitis. Another relevant finding in our work was the presence of advanced features of chronic pancreatitis such as calcifications or ductal dilation (median duct diameter 10 mm) in patients with early disease. This finding may be associated with asymptomatic or subclinical parenchymal injury and inflammation, the reasons for this type of presentation is still unknown but could be related to the patient's pain threshold [26].

According to Yadav et al. [21] and Shah et al. [27], alcohol consumption is related to a more aggressive inflammatory response with increased morbidity, hospital length of stay, and intraoperative complications in patients with chronic pancreatitis who underwent Frey procedure, alcohol and tobacco consumption should be suspended in these patients [13]. Pain control is a cornerstone in the treatment of chronic pancreatitis. Alcohol consumption and smoking habits are contributing factors for incomplete pain relief after the Frey procedure [28]. In this study, a reduction of 50% of pain according to visual analog score was reached; data that is comparable to the one reported by Li et al., who in a retrospective study that included 75 patients evidenced a reduction of 65% in the majority of the population [29]. The same author identifies alcohol consumption as a risk factor for uncontrolled pain after Frey’s procedure, with an OR 7.7 P-value of 0.002 [28]; in our population, this outcome cannot be measured due to the difference in etiologies between regions.

Evidence regarding surgical vs. endoscopic management is sparse. Cahen et al. performed a randomized controlled trial in 19 endoscopic management vs. 20 surgery cases. Pain control was significantly improved with surgery compared to endoscopic treatment (75% vs. 32% pain reduction, P = 0.007 [28]. On the other hand, a more recent retrospective analysis in China found no difference between endoscopic treatment and surgery concerning pain recurrence and hospital stay, but surgery was associated with higher morbidity and complications [29, 30]. In the most recently published guidelines of the American College of Gastroenterology, surgery was recommended in patients with obstructive CP over endoscopy therapy if this tool was unsuccessful or exhausted (strong recommendation, moderate quality of evidence) [10].

Moreover, the United European Gastroenterology guidelines recommend early surgery over surgery at a more advanced stage in terms of long term pain relief, improved quality of life and risk of postoperative exocrine insufficiency but as a weak agreement (grade 2C) [22].

The complication rate in this work at 90 days of the postoperative period was 38.8% (n = 7); they were graded as type I in 85.71% of the cases (n = 6) and type V in 14.29% (n = 1) of the cases; similar data were reported by Gestic et al. and Ray et al. who report a morbidity rate that ranges between 28.7 and 31% respectively [18, 31–34].

The pancreatic leak was identified in 4 patients, and 100% of the cases were classified as biochemical leaks; data related to the one reported by Gestic et al., who reported a 6.8% type B pancreatic fistula rate [7, 33]. Gastrointestinal bleeding is one of the most feared complications that could appear between 2.8–8.2% in some cases; the main causes are the formation of pseudoaneurysms [30, 31, 33] and clinically manifestation with hemorrhagic shock and death or due to anastomosis bleeding. In our population, upper gastrointestinal bleeding was documented in 16.6% of cases, however, it self-resolved, without hemodynamic repercussions or vascular complications. Re-intervention rate could vary between 2–9%, according to Ray et al. [18], and it’s explained in most cases by shock-related bleeding [34, 35]; in this population, reintervention was performed in 1 patient due to upper gastrointestinal bleeding.

Endocrine pancreatic insufficiency is a frequent late complication [1, 2, 33]. 8–34% of de-novo diabetes diagnoses after FP in a medium/long-term follow-up have been described [24, 31, 33]. Our results show a late postoperative diagnosis rate of diabetes mellitus (> 30 days) in 22.2% (n = 4) of the patients who were taken to the PF in a mean follow-up of 42.5 months; nonetheless, any patient required insulin replacement.

Chronic pancreatitis is a known risk factor for pancreatic malignancy and is accepted as a premalignant condition [4, 32]; however, studies are lacking in this regard [19, 20, 22, 25]; according to Kirkegård et al. there is a 7.9-fold increased risk in the first 2 years of follow-up, and through the years, this risk decreases [32]. For that reason, a long-term follow-up for patients with CP confirmed diagnosis is recommended, and in patients in the post-operative period of Frey’s procedure [32, 33]. Ray et al. [18] identified a 4.34% rate of pancreatic malignancy after 44 months of follow-up; data that is comparable with our population in which six patients developed pancreatic cancer; in the majority of the cases, histopathology evidenced was pancreatic adenocarcinoma, with a mean time of presentation of 28 months after the CP procedure was performed; time-lapse of high risk of malignancy growth as claimed by Kirkegård et al. [32]. Our study also shows an association between pancreatic duct dilatation prior to surgery and malignancy.

Among the limitations of our study are the retrospective nature and small sample size. However, the strengths of our study are long-term follow-up that allows us to diagnose a high number of patients with oncologic conditions related to chronic pancreatitis.

## Conclusion

According to our data, Frey’s procedure remains safe and feasible, with acceptable outcomes in terms of pain relief and pancreatic function. The study confirms the importance of a longstanding follow-up due to an inherent risk of pancreatic malignancy. Our data suggest that pancreatic duct size could be related with the malignancy diagnosis after Frey’s procedure; however, further prospective studies with a larger sample size would be helpful to confirm these results.

## Data Availability

The datasets generated and/or analyzed during the current study are not publicly available due to institutional pólices but are available from the corresponding author on reasonable request.

## Abbreviations

CP: Chronic pancreatitis
PD: Pancreatoduodenectomy
HIPAA: Health Insurance Portability and Accountability Act
iCMJE: International Committee of Medical Journal Editors
IRB: Ethics and Research Institutional Committee
HPB: Hepatobiliary and pancreatic surgery
IQRs: Interquartile ranges
BMI: Body mass index
T2DM: Type 2 diabetes mellitus
AP: Acute pancreatitis
